# A Systematic Review of Economic Evidence of Cardiovascular Interventions in India

**DOI:** 10.2174/011573403X309363240730095253

**Published:** 2024-07-30

**Authors:** Saba Abidi, Anandita Nair, Rakhi Ahuja, Shridhar Dwivedi, Sushama Talegaonkar

**Affiliations:** 1 Department of Pharmaceutics, School of Pharmaceutical Sciences, Delhi Pharmaceutical Sciences and Research University, New Delhi, India;; 2 Department of Biological Sciences, Birla Institute of Technology and Sciences, Pilani, India;; 3 School of Allied Health Sciences and Management, Delhi Pharmaceutical Sciences and Research University, New Delhi, India;; 4 Department of Cardiology, National Heart Institute, New Delhi, India

**Keywords:** Economic evaluation, cost-effectiveness analysis, cardiovascular diseases, health interventions, pharmacological interventions, cardiovascular interventions

## Abstract

**Background:**

Cardiovascular diseases (CVDs) continue to be the primary cause of mortality globally and invariably in India as well. The rapid upsurge in the prevalence of CVDs in India has created a pressing need to promote contemporary, sustainable, and cost-effective interventions to tackle the CVD burden. This systematic review integrates the research-based evidence of the cost-effectiveness of various interventions that can be adapted to control CVDs in India.

**Methods:**

Databases, namely, PubMed, Cochrane Library, Embase, and Google Scholar, were searched for data on the economic evaluation of interventions targeting CVD based on the Indian population for a period of 30 years (1991-2021). Two reviewers assessed the articles for eligibility, and data were extracted from the shortlisted articles as per a predefined template, including the quantification of methodological aspects.

**Results:**

In total, 1249 studies were examined, out of which 23 completely met the inclusion criteria for full-text review. A total of 16 studies were based solely on the Indian population, while the rest (7) included South Asia/Asia for the intervention, of which India was a participant nation. Most of the economic evaluations targeted treatment-based or pharmacological interventions (14) for CVDs. The evaluations were based on Decision-based models (10), Randomized controlled Trials (RCTs) (9), and Observational studies (4). The cost-effectiveness ratio for the included studies exhibited a diverse range due to variations in methodological approaches, such as differences in study settings, populations, and inconsistencies in study design. The mean ICER (Incremental Cost-effectiveness ratio) for primordial and primary preventions was found to be 3073.8 (US $2022) and 17489.9 (US $2022), respectively. Moreover, the combined mean value for secondary and tertiary prevention was 2029.6 (US$2022).

**Conclusion:**

The economic evidence of public health interventions are expanding, but their focus is restricted towards pharmacological interventions. There is an urgency to emphasize primordial and primary prevention for better outcomes in health economics decision-making. Technology-based avenues for intervention need more exploration in order to cater to a large population like India.

## 1 INTRODUCTION

Cardiovascular diseases (CVDs) remain to be the predominant cause of mortality across the globe and in India as well. The prevalence of CVDs in India was estimated to be 54.5 million in 2016. Presently, CVDs account for one in four deaths in India, and ischemic heart disease, along with stroke, account for more than 80% of this mortality [1].

CVDs also pose an economic threat for India as the deaths due to CVD, especially targeting the working-age group (25 to 64 years), resulted in the loss of 9.2 million possibly productive and healthy years of life in 2000, and it is anticipated to reach a loss of 17.9 million years by 2030 [2].

The World Bank estimates that India lost 18.2 million DALYs (disability-adjusted life years) because of CVDs in 2010, and in the next 20 years, this loss is projected to reach twice this number [3].

Interventions at every level of prevention (primordial, primary, secondary, and tertiary) have proved to be effective in controlling cardiovascular diseases. But, there is a need for economic evidence of these interventions for low-income countries, such as India.

As there is a rapid increase in the prevalence of CVDs in India, there is an essential requirement for the promotion of cost-effective measures to tackle CVDs. Researchers have endeavored to evaluate these interventions economically in LMICs, and those who have done so have targeted non-communicable diseases as a whole. Recent literature suggests a surge in requirements from healthcare policy-makers for research-based evidence to make appropriate decisions; therefore, there is a need to conduct and assimilate real-world evidence research, which will ultimately help in precise resource allocation [4]. Hence, it is essential to economically evaluate the public health interventions for CVDs as the out–of–pocket expenditure for CVDs is quite high, and the country’s health care resources are not competent enough to tend to these overwhelming costs. Arguably, this is the first systematic review that has integrated the economic evidence of potential interventions pertaining to CVD, specifically in India.

The studies related to the cost-effectiveness of interventions to control CVDs in India were systematically reviewed. The main objectives of the paper were (1) to summarize the cost-effectiveness analysis of interventions to reduce the CVD burden, (2) to synopsize various strategies that have been evaluated in India to control CVDs, (3) to condense the varied methodologies used by different studies to conduct the economic evaluation.

## 2 METHODS

**Fig. (1):**
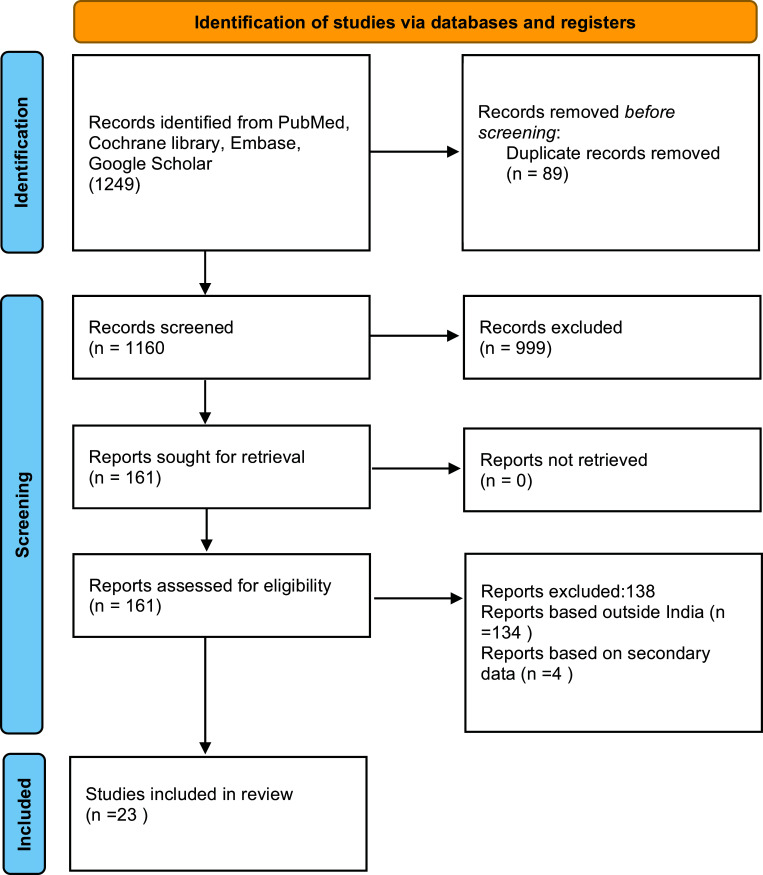
PRISMA for identification of studies which were included in the systematic review. *From*: Page MJ, McKenzie JE, Bossuyt PM, Boutron I, Hoffmann TC, Mulrow CD, et al. The PRISMA 2020 statement: an updated guideline for reporting systematic reviews. BMJ 2021;372:n71. doi: 10.1136/bmj.n71 For more information, visit: http://www.prisma-statement.org/.

The protocol of this review has been registered at PROSPERO (Protocol no. CRD CRD42021274261). The review followed the guidelines from Preferred reporting items for systematic reviews and meta-analyses (PRISMA) (Fig. **1**).

### 2.1 Search Strategy and Selection Criteria

PubMed, Cochrane Library, Embase, and Google Scholar were explored for primary studies on economic evaluation of interventions for cardiovascular diseases from 1991 to 2021 using a predefined vocabulary.

Various arrangements of the following terms were used.

“Cost” OR “ Cost analysis” OR “cost-effectiveness” OR “cost-effectiveness analysis” OR “cost–benefit analysis” OR “economic evaluation” OR “Cost-utility” AND “Primordial prevention” OR “Legislation” OR “Tobacco control” OR “Smoking Legislation” OR “Primary prevention” OR “Health education” OR “Mass education” OR “Physical Activity” OR “Dietary Intake” OR “Dyslipidemia treatment” OR “Hypertensive treatment” OR “Secondary prevention” OR “treatment” OR “surgery” AND “tertiary prevention” AND “Cardiovascular diseases” OR “Heart diseases” OR “Heart failure” OR “Ischemic heart diseases” OR “Cardiac diseases” AND “India” OR “Indian population” ‘were used as MeSH terms.

### 2.2 Selection Criteria

#### 2.2.1 Population and Study Design

The inclusion criteria involved (a) primary studies analyzing any type of intervention targeted to prevent the onset of complications of CVDs, (b) studies based on the Indian population, (c) studies conducting economic evaluation, and (d) studies that used some decision models to assess the cost-effectiveness. Studies including other non-communicable diseases were excluded. This systematic review also excluded news items, reviews, systematic reviews, conference papers, and studies of a country other than India.

#### 2.2.2 Interventions

All types of interventions targeting the prevention of the onset and complications of cardiovascular diseases were included in the systematic review. Primordial, primary, secondary, and tertiary prevention, separately or in combination, were included in the review. Any population-based intervention, such as legislation, smoking bans, tobacco bans, or advertisement bans, was included in primordial prevention. In primary prevention, health education, physician advice, screening, *etc*., were included. Interventions involving the treatment of cardiovascular diseases were included in the secondary and tertiary prevention of cardiovascular diseases.

#### 2.2.3 Types of CVDs

The following diseases were included in the review, namely, coronary artery diseases, ischaemic heart disease, angina and myocardial infarction, congestive heart failure, hypertension.

#### 2.2.4 Types of interventions

1. **Primordial:** Legislation, Tobacco Control, Smoking Legislation
2. **Primary:** Health education, Mass Education, Physical Activity, Dietary Intake
3. **Secondary:** Dyslipidemia treatment, Hypertensive treatment
4. **Tertiary:** Percutaneous Coronary Intervention, Percutaneous Transluminal Coronary Angioplasty, Coronary Artery Bypass graft, Surgery.

#### 2.2.5 Comparators

Every intervention was compared to either another intervention or no intervention.

#### 2.2.6 Outcomes

The health economic outcomes searched in the study were DALYs (disability-adjusted life years), QALYs (quality-adjusted life years), and YLS (years of life saved). Moreover, the cost effectiveness of any intervention was measured using incremental cost-effectiveness ratios (ICERs).

### 2.3 Data Extraction

The databases were searched for the required articles, post searching, and one reviewer (SA) searched and excluded titles that were duplicates. Then, the titles and abstracts of the remaining studies were independently scrutinized by two reviewers (SA and AN). The eligible studies were gone through, and their inclusion or exclusion was decided. Any dispute was solved by discussion. Later, the CHEERS (Consolidated Health Economic Evaluation Reporting Standard) checklist was used to appraise the economic evaluations [5].

After the studies were selected for full-text review, a predesigned template was used to extract the relevant data. The template comprised of the name of the author, publication year, study setting, study design, type of economic evaluation, target population, time horizon, incremental cost-effectiveness ratio, intervention, comparator, economic perspective, methodology, discount rate, sensitivity analysis performed, and evaluated ICERs. The funding source was identified from the statements of acknowledgment or declarations.

### 2.4 Risk of Bias Assessment

As a final step, the CHEERS checklist was used, and the risk of bias was calculated for this systematic review. This checklist has been predominantly used in scrutinizing any economic evaluation, and 22 biases are included, of which 12 are found specifically related to economic studies [5]. Accordingly, the included studies were evaluated, namely, study perspective, explanation about comparators, discount rates used for cost and outcome, target population, evaluated ICERs, sensitivity analysis performed, and description of funding sources and any other conflict of interest.

### 2.5 Quality Assessment of RCTs

A total of 8 RCTs were included in the review. The quality assessment of these RCTs was done using Cochrane Collaboration's tool for assessing the risk of bias [6]. The risk of bias was assessed on the basis of adequate sequence generation, allocation concealment, blinding, incomplete outcome data addressed, free of selective reporting, and free of other biases. The checklist for the quality assessment of the RCTs is provided in the supplementary material.

### 2.6 Currency Conversions

Firstly, the unit costs were converted into US dollars (US$) to have a uniform intercountry comparison. Local currencies were converted into US$ using the stated year of currency conversion, or base year for prices or article publication year. Secondly, costs in US $ were then inflated to 2022 values using the US $ Consumer Price Index for all urban consumers (CPI-U) [7, 8].

## 3 RESULTS

Titles of 1249 articles were searched, of which 197 articles were reported to be eligible for review after removing the duplicates. The search came down to 23 economic evaluations of interventions for the prevention of cardiovascular diseases in India (Fig. **1**).

### 3.1 Study Characteristics

#### 3.1.1 Assessment of Risk of Bias

CHEERS checklist was incorporated to assess the risk of bias for economic evaluation. About 43.5% of studies had all components of the checklist in their model. About 39.1% of the studies did not mention only one aspect of the checklist, such as ICER reported, conflict of interest, time horizon, *etc*. The rest of the studies (17.4%) did not mention two or more components of the economic evaluation. The quality of the RCTs was assessed with the help of the Cochrane tool. (Supplementary material) (Tables **1** and **2**).

#### 3.1.2 Study Design

The majority of the studies (43.5%) used a decision-based model to evaluate the cost-effectiveness of the intervention. Nine studies (39.1%) used the randomized controlled trial, and four studies (17.4%) were observational. Out of RCTs, one study [9] used the Use of Multidrug Pill In Reducing cardiovascular Events (UMPIRE) trial to evaluate the effectiveness of polypill. Individuals or populations at risk or people who have established cardiovascular diseases were taken as the targeted population in all the studies. All types of interventions (Policy level, group level, or population level) were evaluated and compared with no intervention scenario or any active comparator. [3].

#### 3.1.3 Economic Perspective

Eleven studies (47.8%) gave results from the healthcare provider perspective, *i.e.,* direct costs expended by the healthcare system. Three studies took a patient perspective (Out-of-pocket expenditure by patient), and three studies took a societal perspective. Two studies [10, 11] used both patient and healthcare provider perspectives for analysis. Four studies did not state their perspective.

#### 3.1.4 Economic Evaluation

A significant number of studies (69.6%) used cost-effectiveness analysis to evaluate the interventions economically. Six studies (26.1%) used cost-consequences analysis (CCA), and only one study [11-14] used cost-minimisation analysis to establish the effectiveness of different preventive strategies. Some studies took the healthcare provider perspective, and some studies took a societal perspective.

#### 3.1.5 Outcome Measure

The majority of the studies (69.6%) reported their outcomes in terms of 'life-years gained' or 'QALYs' or 'DALYs' in their analysis. The remaining seven studies reported either of the intermediate outcome measures like cost for treatment, number of days in hospital stay, frequency of hospitalization, reduction in blood pressure (BP), BMI (Body Mass Index) and cholesterol, cost per major coronary event averted and any CVD event averted. These intermediate outcome measures are easier to quantify but challenging to conduct a comparison between different interventions.

#### 3.1.6 Discount Rate

Most of the studies (52.2%) assumed a 3% discount rate for costs and effects. In 30.4% of the studies, the discount rate was not applicable. In three studies, no discount was applied.

#### 3.1.7 Funding

Most of the studies (60.8%) mentioned the funding for their research. Almost all studies were funded by public sponsorship like Fogarty International Centre, the National Institutes of Health, Bill and Melinda Gates Foundation, WHO, and World Bank. One study [12] was sponsored by a pharmaceutical company. Only one study [13] was funded by an Indian Organization, the Public Health Foundation of India. This depicts that national funding agencies and decision-makers do not focus on these types of evaluations. A large number of studies (39.1%) did not state funding for their research.

#### 3.1.8 Time Horizon

All studies mentioned the time horizon assumed in their analysis. Nearly half of the studies (43.5%) assumed lifetime as the analytical time horizon. In the remaining studies, the time period varied according to the study design, varying from 5-7 days to 10, 20, and 50 years.

### 3.2 Studies on Primordial Prevention

Four studies evaluated the effectiveness of primordial prevention consisting of a combination of taxation on alcohol laws, clean indoor advertisement ban, and labeling [14-19]. Alcohol control is an important intervention for cardiovascular diseases. A combination of several primary interventions was identified as more cost-effective than a single intervention [14]. One study [15] concluded that only taxation on alcohol was not found to be resourceful, but taxation, along with the advertisement ban, was calculated as more cost-effective. The complete ban on smoking in public places was identified as cost-saving, and many life-years were gained, and events of acute myocardial infarction were averted pertaining to this ban [18]. A cessation of smoking program based on a school was evaluated and found to be effective [19].

The mean (range) incremental cost-effectiveness ratio (ICER) of the interventions for primordial prevention was found to be 3073.8 (US$2022) (65.5, 10870) based on the type and number of interventions implemented and the coverage of these interventions (Table **3**).

### 3.3 Studies on Primary Prevention

The primary interventions were evaluated for cost effectiveness by five studies. A study analyzed the outcome of expanding national insurance to cover the primary prevention of cardiovascular diseases, and this intervention was reported to be cost-effective [20-22]. Other policy-based strategies, such as nicotine replacement therapy in the tobacco control program, were also found to be economical [21-23]. Physician counseling or recommendations on a proper basis were found to be cost-effective when compared to the absence of intervention [15, 16].

An important primary intervention, which was found to be economical, was a salt reduction in the diet. This intervention was analyzed in comparison with a combined strategy of salt reduction, legislation, and public education campaigns. This combined component was found to be productive [14, 16, 17]. Another dietary intervention involving the reduction of trans fat was found to be cost-wise beneficial. Either media campaigns to reduce trans fat in the diet or the intake of polyunsaturated fatty acids was found to be an effective cost-reduction strategy with respect to the DALY averted [16].

The mean [range] ICER of primary preventions was calculated as 17489.9 (US$2022) (40,140388.2), which included various interventions ranging from dietary interventions to school health education programs.

### 3.4 Studies on Secondary and Tertiary Prevention

Many studies (15) conducted the economic evaluation of the pharmacological interventions. A study analyzed the consequences of a policy intervention in which the extension of national insurance coverage for secondary and tertiary prevention was evaluated as cost-effective when compared to no intervention and less cost-effective when compared to primary prevention [23]. Other policy interventions, such as the expansion of drugs to treat acute myocardial infarction, were stated to be cost-effective [24].

Among pharmacological interventions, multidrug treatment provided to those who have more than 35% cardiovascular risk was more cost-effective when compared with >5% cardiovascular risk [14]. Multidrug therapy as a blood pressure-lowering intervention was found to be the most cost-effective in people with more than 35% cardiovascular risk [25]. A study compared treat-to-target strategies and benefit-based tailored treatment to lower CVD risk and found the latter to be the most cost-effective [20]. Two studies have reported that diuretics-based hypertensive treatment is cost-effective [17, 26]. A decision support system was also reported to be cost-effective when compared to chart-based support for hypertension management [13]. A study evaluated the rationale of prehospital ECG as a basis for accurate referral and reported it to be cost-effective when compared to no ECG-based referral [ICER-5620 (US2022)] [22].

The ICERS for secondary and tertiary levels of prevention were found to range between 2.4 (US$2022) to 13850 (US$2022) with a mean of 2029.6 .The diversity in the values of ICER was based on the type of intervention, type of population, the coverage, and the duration of the interventions.

### 3.5 Studies with Combined Interventions

Three studies evaluated the combination of various interventions and identified the most cost-effective one. A study [15] evaluated a combined intervention that included taxation, advertisement ban, and physician’s advice and found it to be cost-effective for reducing the consumption of alcohol. For controlling blood pressure, the combination of salt legislation, health education, and clinical treatment of patients with more than 35% cardiovascular risk with an expanded drug regime, including statins, diuretics, beta-blockers, and aspirin, was reported to be very cost-effective [17, 25].

## 4 DISCUSSION

This review revealed that the majority of interventions targeting cardiovascular diseases were cost-effective. However, the strength of cost-effectiveness of every intervention varied across the types of study design (RCTs, decision-based model, observational studies). The most economical interventions were those that targeted the population level. This suggests that population-level healthcare modalities should be analyzed and implemented by national decision-makers to reduce the burden of cardiovascular diseases. Primordial preventive strategies like salt legislation, food advertisement ban, food labeling, taxation, *etc*. should be emphasized.

Primary and secondary preventions were also reported to be cost-effective, such as multidrug therapy for reducing cardiovascular risk and hypertensive treatment with diuretics such as blood pressure-lowering strategies. Policymakers can explore these strategies in order to reduce the cardiovascular disease burden in India. The comparators were also the basis of variation in the results when evaluating the cost-effectiveness of interventions. When an intervention was compared to the no-intervention scenario, this situation is based on no costs and no effect, which is unlikely to happen in any country. So, this reason needs to be further addressed. When the comparators were not mentioned for any intervention, it led to inaccurate cost-effectiveness analysis.

This review also identified a number of studies conducting cost-effectiveness analysis based on WHO-CHOICE (CHOosing Interventions that are cost-effective) programs and DCPP2 (Disease Control Priorities in developing countries) [11, 21, 25, 27-35]. These strategies have been made collectively for South Asian regions, of which India is a part. Indian researchers and organizations need to do more evidence-based research to identify cost-effective strategies specifically for India.

Although almost all interventions have been targeted in this systematic review, no study has been conducted on the role of increased physical activity in reducing cardiovascular risk. This can be explored in the subsequent research. Studies have evidenced the role of physical activity in improving the prognosis of patients with CVDs [36, 37]. The mental health of patients with CVDs was also not considered in any study because good mental health has also been proven to improve the clinical outcomes of the patients. Cognitive behavioral therapy has been demonstrated to be an effective treatment for improving the mental health of patients with CVDs and, hence, can be used in clinical care to improve health outcomes [38].

Public health technology can be utilized to cater to a large population like India [36]. The investment in technology-based intervention can prove to be economical. These technical interventions need to be explored more, and established interventions need to be proven cost-effective for India. Moreover, non-invasive techniques could be explored to identify individuals who do not need significant interventions to prevent CVDs [39, 40]. One of these techniques is the modified Haller index, which identifies individuals with a low pre-test probability of coronary artery diseases [41]. These techniques reduce the need for unnecessary examinations, ultimately reducing the economic burden on healthcare resources.

This review is rare as it involves all types of interventions at all levels (primordial, primary, secondary, and tertiary) to reduce the burden of CVDs in India. This review has cogitated all possible interventions to control CVDs. This review can assist policymakers to ponder different strategies both individually and in combination, for policymaking. Moreover, the economic evaluation of interventions can help the decision-makers to appropriately allocate assets in a resource-constrained country like India.

This review has limitations as well. Unpublished and grey literature has not been included in the review. This review has also excluded studies based on secondary data. Studies of South Asia, which included India, have been evaluated in this review, but the studies specifically on the Indian population would have given a clearer deduction.

Therefore, our review highlights the scarcity of high-quality economic evaluations to ascertain the most cost-effective strategies to reduce the CVDs burden in India.

## CONCLUSION

The published literature has given economic evidence of the role of interventions in reducing the burden of cardiovascular diseases in India. This research-based evidence would provide insights for policymakers regarding the appropriate allocation of resources to reduce the socioeconomic burden of CVDs on Indian healthcare systems. The economic evidence of public health interventions is increasing, but they are mainly centered towards treatment interventions. There is a need to emphasize primordial and primary prevention for better health and economic decision-making. Technology-based interventions need to be explored more to cater to a large population like India. Lastly, more research is needed to evaluate these interventions economically.

## Figures and Tables

**Table 1:** Characteristics of the studies included in the systematic review.

**Characteristics**	**Studies, n**
**Interventions**	
Primordial	3
Prevention	4
Pharmacological	9
Surgical	5
Combined	2
**Perspective**	
Patient	3
Healthcare	16
Societal	3
Combined	1
**Study Design**	
Randomized Controlled Trials	9
Decision based model	10
Observational	4
**Type of Economic evaluation**	
Cost Effectiveness Analysis	16
Cost comparison/consequence analysis	6
Cost Minimisation Analysis	1
**Discount rate applied**	
3%	12
No discount applied	3
Not applicable	7
Not clear	1
**ICER reported**	
Yes	21
No	2
**Sensitivity Analysis done**	
Yes	15
No	4
Not Applicable	4
**Time horizon**	
<1 year	7
>1 year	11
Not applicable	4
Not clear	1
**Published period**	
1991-2001	4
2002-2011	8
2012-2021	11

**Table 2:** Methodological aspects of the studies included in the systematic review of economic evaluations of interventions for prevention and control of cardiovascular diseases in India.

**S. No.**	**Authors Name**	**Country**	**Population**	**Intervention**	**Comparator**	**Economic Perspective**	**Methodology**	**Outcome Measure**
1	Ahuja *et al*, 1997 [26]	India	Patients with mild hypertension Consecutive patients with mild essential hypertension, diastolic BP 90 to 110 mmHg, age 35 to 70 years.	Antihypertensive regimens with diuretics	Antihypertensive regimens without diuretics	Patient	RCT-based CEA	Mean cost of control of BP to target levels per patient per day in control and study groups
2	Anchala *et al*, 2015 [13]	India	Patients with hypertension (30Ð64 years)	Decision support system for hypertension management	Chart-based support for hypertension management	Healthcare provider	RCT-based CEA	Cost per unit reduction in SBP
3	Basu *et al*, 2015 [20]	India	Population at risk of CVD and with existing CVD	Expansion of national insurance to cover primary prevention, secondary prevention and tertiary treatment for CVD	Active comparators	Healthcare provider	Decision model-based CEA	Cost of treatment/prevention strategies coverage per DALY averted
4	Basu *et al*, 2016 [23]	India	Individuals aged 30Ð70 years at high CV risk (³10%)	A treat-to-target strategy emphasizing lowering blood pressure to a target, A benefit-based tailored treatment strategy emphasizing lowering CVD risk, A hybrid strategy currently recommended by the WHO	Active comparators	Healthcare provider	Decision modeling-based CEA	DALYS averted by reducing CVD deaths
5	Brown *et al*, 2013 [19]	India	School students: aged 14 years and above	Project MYTRI Four intervention components: 1. classroom activities/behavioral interventions 2. peer-led health activism 3. posters 4. parent cards	No intervention	Societal	RCT-based CCA	QALYs gained by averted smoking and medical costs
6	Cecchini *et al*, 2010 [16]	South Asia region (India)	Population-based and individuals at high risk (BMI≥25 kg/ m2, high BP, cholesterol, diabetes)	Dietary and physical activity interventions targeted at: 1. school level 2. worksites 3. mass media campaigns 4. fiscal measures 5. physician counseling 6. food advertising regulation 7. food labeling	No intervention	Healthcare provide	Decision model-based CEA	Reduction in BMI, cholesterol, SBP, fat intake and increase in bre consumption
7	Chisholm *et al*, 2004 [15]	South Asia region (India)	Individuals at risk of alcohol and tobacco use	Interventions to reduce use of alcohol and tobacco use	Various	Healthcare provider	Decision model-based CEA	DALYs averted by reducing use of tobacco, alcohol and illicit drug
8	Donaldson *et al*, 2011 [18]	India	Individuals at risk of secondhand smoking	Prohibition of smoking in public places	No smoking ban	Societal	Decision model-based CEA	Life years saved and QALYs gained by complete smoking ban in public places and by averted AMI
9	Lamy *et al*, 2014 [28]	Asia (India)	Patients requiring revascularisation procedure	Off-pump CABG	On-pump CABG	Healthcare provider and patient	RCT-based CMA	Cost per patient in the off- pump CABG *vs* on-pump CABG group
10	Lin. K *et al*, 2019 [27]	China, India, Mexico, Nigeria, South Africa	Adults aged 30-84 yrs with ACD	Secondary prevention by Fixed-dose combination pill/Poly pill (aspirin, lisinopril, atenolol, simvastatin)	Poly pill is compared with other prescribed drug for ACD	Healthcare sector	Micro simulation Markov model CEA	averted adverse CV events, DALYs ,and the no. of patients who would need to be treated for 10 years to reduce one major adverse CV event with the polypill compared with current care.
11	Malhotra *et al*, 2001 [30]	India	Patients with unstable angina	Enoxaparin	UFH	Healthcare provider	RCT-based CCA	Mean cost per patient in UFH and enoxaparin groups
12	Megiddo *et al*, 2014 [24]	India	Patients with acute myocardial infarction	Policies that expand the use of aspirin, injectable streptokinase, beta-blockers, ACE inhibitors, and statins for treatment and secondary prevention of AMI	Active comparators	Healthcare provider	Decision model-based CEA	DALYS averted by expanding use of CVD prevention drugs
13	Murray *et al*, 2003 [17]	South Asia region (India)	High CV risk individuals	Behavioral interventions and treatment strategies to lower SBP and cholesterol	Various	Healthcare provider	Decision model-based CEA	DALYs averted by reduction in CVD risk
14	Namboodiri *et al*, 2004 [31]	India	Patients awaiting pacemaker implant	DDD *vs* VDD pacemakers	nil	Not stated (direct medical costs)	Observational study-based CCA	Costs compared *vs* clinical efficacy and complications between two arms
15	Nanjappa *et al*, 1998 [32]	India	912 patients with symptomatic rheumatic mitral stenosis	Transvenous mitral commissurotomy: double- lumen (Accura) variable- sized single balloon	Triple-lumen (Inoue) balloon	Not stated (direct medical costs)	Observational study-based CCA	Costs compared *vs* haemodynamic stability in both arms
16	Ortegon *et al*, 2012 [14]	South Asia region (India)	Population-based and individuals at high CV risk	123 single or combination prevention and treatment strategies for CVD, diabetes and smoking	Various	Healthcare provider	Decision model-based CEA	DALYs averted by reducing CVD, diabetes and tobacco related disease
17	Patel *et al*, 2014 [33]	India	Patients with hypertension	Nebivolol (2.5mg, 5mg, 10 mg)	Sustained release metoprolol succinate (25mg, 50mg, 100mg)	Patient	RCT-based CEA	ICER per unit reduction in blood pressure per day
18	Sanmukhani, *et al*, 2010 [12]	India	Patients at risk of CVD (primary prevention) Patients with history of CVD (secondary prevention)	Simvastatin 40 mg Pravastatin 40 mg	No therapy	Patient	Observational study-based CEA	Cost per major coronary event averted , Cost per CHD death averted
19	Schulman-Marcus *et al*, 2010 [22]	India	Patients with acute coronary syndrome	Prehospital ECG performed by a GP to improve timely access to reperfusion by accurate referral to a hospital	ECG-based diagnosis *vs* no ECG tests in acute chest pain	Societal	Decision model-based CEA	QALY gained by accurate referral to hospital in patients with ACS
20	Sengottuvelu *et al*, 2016 [34]	India	65 patients requiring angiogram followed by fractional flow reserve	Fractional flow reserve	Angiography	Not stated (direct medical costs)	Observational study-based CCA	Costs compared *vs.* management decision
21	Shafiq *et al*, 2006 [10]	India	Patients with unstable angina	Low molecular-weight heparinsÑenoxaparin, nadroparin and dalteparin	Active comparators	Patients and healthcare provider	RCT-based CEA	ICER per MACE outcomes (MI, recurrent angina, death)
22	Singh *et al*, 2018 [9]	India	1000 individuals aged ≥18 years, both sexes with high risk of CVD (≥15% CVD risk over 10 years).	Secondary prevention by 2 versions of the polypill compared with usual care during CVD	Polypill compared with usual care groups in UMPIRE from the health sector prospective.	health sector perspective.	UMPIRE trial CEA	The mean cost per capita patient significantly lower with the polypill strategy (-$203 per person.(95%Cl: -286,-119, *p*<0.01).
23	Turi *et al*, 1991 [35]	India	40 patients with severe rheumatic mitral stenosis	Percutaneous balloon commissurotomy	Surgical closed commissurotomy	Not stated (direct medical costs)	RCT-based CCA	Costs compared *vs.* haemodynamic stability in both arms

**Abbreviations:** RCT-Randomised Control Trial, CEA-Cost Effectiveness Analysis, CUA-Cost Utility Analysis, CBA- Cost Benefit Analysis, NA- Not Applicable, ICER-Incremental Cost Effectiveness Ratio, WHO- World Health Organisation, DCP-Disease Control Priorities in Developing Countries., CVD- Cardiovascular Diseases, QALY, quality-adjusted life years; DALY, disability-adjusted life years, BMI-Body Mass Index.

**Table 3:** Interventions and comparators observed in the studies included in the systematic review of economic evaluations of interventions for prevention and control of cardiovascular diseases in India.

**Intervention**	**Comparator**	**Time Horizon**	**ICER (Incremental Cost Effectiveness Ratio)**	**ICER in US$ Adjusted for Inflation Till 2022**
***Tobacco control strategies***				
Increased taxation (60%) [14]	No intervention	Lifetime	87 (Int$, 2005)	65.5
Tax increase+ clean indoor air law [14]	Increased taxation	Lifetime	156 (Int$, 2005)	117.4
Tax increase+ clean indoor air law +advertisement ban [14]	Tax increase+ clean indoor air law	Lifetime	182 (Int$, 2005)	137
Tax increase+ clean indoor air law +advertisement ban +information/ labeling [14]	Tax increase+ clean indoor air law+ advertisement ban	Lifetime	198 (Int$, 2005)	149.1
Tax increase+ clean indoor air law+ advertisement ban +information/labeling +brief advice to quit [14]	Tax increase+ clean indoor air law+ advertisement ban +information/labeling	Lifetime	4176 (Int$, 2005)	3144.3
Tax increase +clean indoor air law +advertisement ban+ information/labeling +brief advice to quit + counseling to quit [14]	Tax increase +clean indoor air law +advertisement ban +information/labeling+ brief advice to quit	Lifetime	4229 (Int$, 2005)	3184.2
Complete smoking ban in public places [18]	Current legislation for partial smoking ban in public places	10 years	Cost/AMI case averted and Cost/LY gained: Cost Saving	
School-based smoking prevention program [19]	No intervention		Cost/QALY added: 2769, Cost/LY gained: 4348 (US$ 2006)	Cost/QALY added: 6922.5, Cost/LY gained: 10870
***Counselling***				
Physician Counseling [16]	No intervention	20, 50 yrs	6155, 5553 (US$ 2005)	14482.3, 13065.9
***Blood - pressure lowering interventions***				
Diuretics based Hypertensive treatments [26]	Non-diuretics based hypertensive treatments	6 months	Cost effectiveness ratio: 0.21	
Decision support system for hypertension management (DSS) [13]	Chart-based support (CBS) for hypertension management	1 year	cost-effective ratio for CBS and DSS groups was $96.01 and $36.57 per mm of SBP reduction, respectively	
Treatment of SBP above 160 mm Hg with BB and diuretic [17]	No intervention	Lifetime	36 ( Int$, 2000)	84.7
Treatment of SBP above 140 mm Hg with BB and diuretic [17]	No intervention	Lifetime	90 ( Int$, 2000)	140.9
***Salt reduction interventions***				
Salt reduction through voluntary agreements with industry [17]	No intervention	Lifetime	37 ( Int$, 2000)	140.9
Salt reduction *via* legislation +health education *via* mass media [17]	No intervention	Lifetime	17 ( Int$, 2000)	40
Population-wide reduction in salt intake legislation [17]	No intervention	Lifetime	19 ( Int$, 2000)	44.7
***Dietary intervention***				
School based interventions [16]	No intervention	20 yrs	Dominant	Dominant
Worksite interventions [16]	No intervention	20 yrs	6151 (US$ 2005)	14472.9
Mass media campaigns [16]	No intervention	20 yrs	15552 (US$ 2005)	36592.9
Fiscal Measures [16]	No intervention	20 yrs	cost saving	cost saving
Food advertising regulation [16]	No intervention	20 yrs	3186 (US$ 2005)	7496.5
Food labeling [16]	No intervention	20 yrs	952 (US$ 2005)	2240
School based interventions [16]	No intervention	50 yrs	59665 (US$ 2005)	140388.2
Worksite interventions [16]	No intervention	50 yrs	4491 (US$ 2005)	10567
Mass media campaigns [16]	No intervention	50 yrs	8575 (US$ 2005)	20176.5
Fiscal Measures [16]	No intervention	50 yrs	cost saving	cost saving
Food advertising regulation [16]	No intervention	50 yrs	332 (US$ 2005)	781.2
Food labeling [16]	No intervention	50 yrs	776 (US$ 2005)	1825.9
***CVD prevention and treatment***				
Treatment of CHF with diuretics [14]	No intervention	Lifetime	81 (Int$, 2005)	61
Preventive multidrug treatment for >35% risk of CVD event [14]	Treatment of CHF with diuretics	Lifetime	146 (Int$, 2005)	109.9
Preventive multidrug treatment for >35% risk of CVD event + multidrug treatment of post-acute IHD and stroke +diuretics & exercise for CHF [14]	Treatment of CHF with diuretics	Lifetime	152 (Int$, 2005)	114.4
Preventive multidrug treatment for >35% risk of CVD event + multidrug treatment of acute MI or post-acute IHD & stroke + diuretics & exercise for CHF [14]	Treatment of CHF with diuretics	Lifetime	404 (Int$, 2005)	304.2
Preventive multidrug treatment for >25% risk of CVD event + multidrug treatment of acute MI or post-acute IHD & stroke + diuretics & exercise for CHF [14]	Treatment of CHF with diuretics	Lifetime	462 (Int$, 2005)	347.8
Preventive multidrug treatment for >5% risk of CVD event [14]	Treatment of CHF with diuretics	Lifetime	1817 (Int$, 2005)	1368.1
Treatment with statins for total cholesterol concentrations above education 6.2 mmol/L [17]	No intervention	Lifetime	47 ( Int$, 2000)	110.6
Treatment with statins for total cholesterol concentrations above education 5.7 mmol/L [17]	No intervention	Lifetime	71 ( Int$, 2000)	167.1
Treatment of SBP above 140 mm Hg with	No intervention	Lifetime	84 ( Int$, 2000)	197.6
BB and diuretics and with statins for total cholesterol concentrations above 6.2 mmol/L [17]				
Multiple drug therapy in >35% CV risk over 10 years [17]	No intervention	Lifetime	NM	NM
Multiple drug therapy in >25% CV risk over 10 years [17]	No intervention	Lifetime	33 ( Int$, 2000)	77.6
Multiple drug therapy in >15% CV risk over 10 years [17]	No intervention	Lifetime	48 ( Int$, 2000)	112.9
Multiple drug therapy in >5% CV risk over 10 years [17]	No intervention	Lifetime	77 ( Int$, 2000)	181.2
Aspirin to baseline [24]	No intervention	Lifetime	0.49 (US$ 2014)	2.4
Aspirin+injection streptokinase [24]	Aspirin to baseline	Lifetime	615 (US$ 2014)	3075
Aspirin to baseline [24]	No intervention	Lifetime	265 (US$ 2014)	1325
Aspirin+BB [24]	Aspirin to baseline	Lifetime	1740 (US$ 2014)	8700
Aspirin+BB+ACEi [24]	Aspirin+BB	Lifetime	2770 (US$ 2014)	13850
Polypill to baseline [24]	Aspirin+BB+ACEi+statin	Lifetime	1690 (US$ 2014)	8450
Polypill (aspirin, statin and two blood pressure lowering drugs) [9]	Usual Care	15 months	75(US$2018)	250
Use of ECG for chest pain [22]	No ECG	-	1124(US$2010)	5620
***National Insurance***				
Insurance coverage for primary prevention of CVD [20]	Status Quo	20 yrs	469 (US$ 2014)	23454
Insurance coverage for secondary prevention of CVD [20]	Status Quo	20 yrs	2404 (US$ 2014)	12020
Insurance coverage for tertiary treatment of CVD [20]	Status Quo	20 yrs	2253 (US$ 2014)	11265
Insurance coverage for primary+secondary prevention of CVD [20]	Primary prevention only	20 yrs	2431 (US$ 2014)	12155
Insurance coverage for primary+tertiary prevention of CVD [20]	Primary prevention only	20 yrs	2241 (US$ 2014)	11205
Insurance coverage for secondary+tertiary prevention of CVD [20]	Primary prevention only	20 yrs	Neg -10728 (US$ 2014)	-53640

**Abbreviations:** ACEi, ACE inhibitors; BB, beta-blockers (blood pressure-lowering agents; CHF, congestive heart failure; CV, cardiovascular; CVD, cardiovascular diseases; DALY, disability-adjusted life years; GDP, gross domestic product;; ICER, incremental cost-effectiveness ratio; QALY, quality-adjusted life years; SBP, systolic blood pressure, CV Risk-Cardiovascular Risk.

## Data Availability

All data generated or analysed during this study are included in this published article.

## LIST OF ABBREVIATIONS

ACEi: ACE Inhibitors
BB: Beta-Blockers Blood Pressure-Lowering Agents
CHF: Congestive Heart Failure
CV: Cardiovascular
CVD: Cardiovascular Diseases
DALY: Disability-Adjusted Life Years

## STANDARDS OF REPORTING

PRISMA guidelines and methodology were followed.
