# Supplementary material for: A Systematic Review of Economic Evidence of Cardiovascular Interventions in India
Source: Curr Cardiol Rev. 2024 Jul 30;20(6):E300724232473. doi: 10.2174/011573403X309363240730095253 (PMC11440333; doi:10.2174/011573403X309363240730095253)
Supplement: Supplementary file 1 — Supplementary material is available on the publisher’s website along with the published article. PRISMA and CHEERS checklist is available on the publisher’s website along with the published article. [file CCR-20-E300724232473_SD1.pdf]

Supplementary Material

A Systematic Review of Economic Evidence of Cardiovascular Interventions in India

Saba Abidi[1], Anandita Nair[2], Rakhi Ahuja[3], Shridhar Dwivedi[4] and Sushama Talegaonkar[1,*]

[1]Department of Pharmaceutics, School of Pharmaceutical Sciences, Delhi Pharmaceutical Sciences and Research University, New Delhi, India; [2]Department of Biological Sciences, Birla Institute of Technology and Sciences, Pilani, India; [3]School of Allied Health Sciences and Management, Delhi Pharmaceutical Sciences and Research University, New Delhi, India; [4]Department of Cardiology, National Heart Institute, New Delhi, India

Assessment of the risk of bias by CHEERS checklist of the studies included in the systematic review for economic evaluation of interventions for prevention and control of cardiovascular diseases in India.

First Author and year	Perspective	Comparator	Target Group	Model type	Time horizon	Cost discounting	Outcome discounting	Conversion rate	Parameters	ICER reported	Conflict of interest	Source of funding declared
Ahuja et al, 1997[26]	+	+	+	+	+	−	+	+	+	+	+	+
Anchala et al, 2015[13]	+	+	+	+	+	+	+	+	+	−	+	+
Basu et al, 2015[20]	+	+	+	+	+	+	+	+	+	+	−	+
Basu et al, 2016[23]	+	+	+	+	+	+	+	+	+	+	+	+
Brown et al, 2013[19]	+	−	+	+	+	−	+	+	+	+	+	+
Cecchini et al, 2010[16]	+	−	+	+	+	+	+	+	+	+	+	+
Chisholm et al, 2004[15]	+	+	+	+	−	+	+	+	+	+	+	+
Donaldson et al, 2011[118]	+	−	+	+	+	+	+	+	+	+	+	+
Lamy et al, 2014[29]	+	+	+	+	+	+	+	+	+	+	+	+
Lin. K et al, 2019[27]	+	+	+	+	+	+	+	+	+	+	+	−
Malhotra et al, 2001[31]	+	+	+	+	+	−	+	+	+	+	+	+
Megiddo et al, 2014[24]	+	+	+	+	+	+	+	+	+	+	+	+
Murray et al, 2003[17]	+	+	+	+	+	+	+	+	+	+	+	+
Namboodiri et al, 2004[31]	−	−	+	+	−	+	+	+	+	+	+	+
Nanjappa et al, 1998[32]	−	+	+	+	−	+	+	+	+	+	−	+
Ortegon et al, 2012[14]	+	+	+	+	+	+	+	+	+	+	+	+

Patel et al, 2014[34]	+	+	+	+	+	+	+	+	+	−	+	+
Sanmukhani, et al, 2010[12]	+		+	+	+	+	+	+	+	+	+	+
Schulman-Marcus et al, 2010[22]	+	+	+	+	+	+	+	+	+	+	+	+
Sengottuvelu et al, 2016[35]	−	+	+	+	−	−	+	+	+	+	+	+
Shafiq et al, 2006[10]	+	+	+	+	+	−	+		+	+	+	+
Singh et al, 2018[9]	+	+	+	+	+	+	+	+	+	+	+	+
Turi et al, 1991[35]	−	+	+	+	+	−	+	+	+	+	+	+

Supplementary Material 2. Quality Assessment of Randomized controlled trials (RCTs).

	Question	Ahuja *et al*	Anchala *et al*	Brown *et al*	Lamy *et al*	Malhotra *et al*	Patel *et al*	Shafiq *et al*	Singh *et al*	Turi *et al*
1	Adequate sequence generation?	no	yes	yes	yes	yes	yes	yes	yes	unclear
2	Allocation concealment?	yes	yes	unclear	yes	unclear	yes	unclear	yes	unclear
3	Blinding	unclear	unclear	unclear	yes	yes	no	yes	yes	no
4	Incomplete outcome data addressed?	unclear	yes	yes	unclear	unclear	unclear	unclear	unclear	unclear
5	Free of selective reporting?	unclear	yes	yes	yes	yes	yes	yes	yes	unclear
6	Free of other bias?	unclear	yes	yes	yes	unclear	yes	yes	yes	unclear

Section and Topic	Item #	Checklist item	Location where item is reported
**TITLE**			
Title	1	Identify the report as a systematic review.	Page 1
**ABSTRACT**			
Abstract	2	See the PRISMA 2020 for Abstracts checklist.	Page 1
**INTRODUCTION**			
Rationale	3	Describe the rationale for the review in the context of existing knowledge.	Page 2
Objectives	4	Provide an explicit statement of the objective(s) or question(s) the review addresses.	Page 3
**METHODS**			
Eligibility criteria	5	Specify the inclusion and exclusion criteria for the review and how studies were grouped for the syntheses.	Page 3
Information sources	6	Specify all databases, registers, websites, organisations, reference lists and other sources searched or consulted to identify studies. Specify the date when each source was last searched or consulted.	Page 3
Search strategy	7	Present the full search strategies for all databases, registers and websites, including any filters and limits used.	Page 3

Selection process	8	Specify the methods used to decide whether a study met the inclusion criteria of the review, including how many reviewers screened each record and each report retrieved, whether they worked independently, and if applicable, details of automation tools used in the process.	Page 4
Data collection process	9	Specify the methods used to collect data from reports, including how many reviewers collected data from each report, whether they worked independently, any processes for obtaining or confirming data from study investigators, and if applicable, details of automation tools used in the process.	Page 4
Data items	10a	List and define all outcomes for which data were sought. Specify whether all results that were compatible with each outcome domain in each study were sought (e.g. for all measures, time points, analyses), and if not, the methods used to decide which results to collect.	Page 3,4
	10b	List and define all other variables for which data were sought (e.g. participant and intervention characteristics, funding sources). Describe any assumptions made about any missing or unclear information.	Page 3,4
Study risk of bias assessment	11	Specify the methods used to assess risk of bias in the included studies, including details of the tool(s) used, how many reviewers assessed each study and whether they worked independently, and if applicable, details of automation tools used in the process.	Page 5
Effect measures	12	Specify for each outcome the effect measure(s) (e.g. risk ratio, mean difference) used in the synthesis or presentation of results.	Page 3,5
Synthesis methods	13a	Describe the processes used to decide which studies were eligible for each synthesis (e.g. tabulating the study intervention characteristics and comparing against the planned groups for each synthesis (item #5)).	Page 3,4
	13b	Describe any methods required to prepare the data for presentation or synthesis, such as handling of missing summary statistics, or data conversions.	Page 5
	13c	Describe any methods used to tabulate or visually display results of individual studies and syntheses.	Page 4,5
	13d	Describe any methods used to synthesize results and provide a rationale for the choice(s). If meta-analysis was performed, describe the model(s), method(s) to identify the presence and extent of statistical heterogeneity, and software package(s) used.	Page 5
	13e	Describe any methods used to explore possible causes of heterogeneity among study results (e.g. subgroup analysis, meta-regression).	-
	13f	Describe any sensitivity analyses conducted to assess robustness of the synthesized results.	-
Reporting bias assessment	14	Describe any methods used to assess risk of bias due to missing results in a synthesis (arising from reporting biases).	Page 5
Certainty assessment	15	Describe any methods used to assess certainty (or confidence) in the body of evidence for an outcome.	-
**RESULTS**			
Study selection	16a	Describe the results of the search and selection process, from the number of records identified in the search to the number of studies included in the review, ideally using a flow diagram.	Figure 1
	16b	Cite studies that might appear to meet the inclusion criteria, but which were excluded, and explain why they were excluded.	Page 6
Study characteristics	17	Cite each included study and present its characteristics.	Table 1
Risk of bias in studies	18	Present assessments of risk of bias for each included study.	Supplementary material
Results of individual studies	19	For all outcomes, present, for each study: (a) summary statistics for each group (where appropriate) and (b) an effect estimate and its precision (e.g. confidence/credible interval), ideally using structured tables or plots.	Table 1 & 2
Results of syntheses	20a	For each synthesis, briefly summarise the characteristics and risk of bias among contributing studies.	Page 6,7
	20b	Present results of all statistical syntheses conducted. If meta-analysis was done, present for each the summary estimate and its precision (e.g. confidence/credible interval) and measures of statistical heterogeneity. If comparing groups, describe the direction of the effect.	Page 8, 9

	20c	Present results of all investigations of possible causes of heterogeneity among study results.	-
	20d	Present results of all sensitivity analyses conducted to assess the robustness of the synthesized results.	-
Reporting biases	21	Present assessments of risk of bias due to missing results (arising from reporting biases) for each synthesis assessed.	Page 5,6
Certainty of evi-dence	22	Present assessments of certainty (or confidence) in the body of evidence for each outcome as-sessed.	-
**DISCUSSION**			
Discussion	23a	Provide a general interpretation of the results in the context of other evidence.	Page 9, 10
	23b	Discuss any limitations of the evidence included in the review.	Page 10
	23c	Discuss any limitations of the review processes used.	Page 10
	23d	Discuss implications of the results for practice, policy, and future research.	Page 10
**OTHER INFORMATION**			
Registration and protocol	24a	Provide registration information for the review, including register name and registration num-ber, or state that the review was not registered.	PROSPERO CRD42021274261
	24b	Indicate where the review protocol can be accessed, or state that a protocol was not prepared.	PROSPERO CRD42021274261
	24c	Describe and explain any amendments to information provided at registration or in the proto-col.	-
Support	25	Describe sources of financial or non-financial support for the review, and the role of the fun-ders or sponsors in the review.	Not funded (Declaration)
Compet-ing in-terests	26	Declare any competing interests of review authors.	No conflict (Declaration)
Availability of data, code and other materials	27	Report which of the following are publicly available and where they can be found: tem-plate data collection forms; data extracted from included studies; data used for all anal-yses; analytic code; any other materials used in the review.	All the studies included for data extraction are available publicly

From: Page MJ, McKenzie JE, Bossuyt PM, Boutron I, Hoffmann TC, Mulrow CD, et al. The PRISMA 2020 statement: an updated guideline for reporting systematic reviews. BMJ 2021;372:n71. doi: 10.1136/bmj.n71

For more information, visit: http://www.prisma-statement.org/
